# Optimal integration of actions and their visual effects is based on both online and prior causality evidence

**DOI:** 10.1038/s41598-018-28251-x

**Published:** 2018-06-28

**Authors:** Nienke B. Debats, Herbert Heuer

**Affiliations:** 1 Department of Cognitive Neuroscience, Universität Bielefeld, Bielefeld, Germany; 2 Cognitive Interaction Technology Center of Excellence (CITEC), Universität Bielefeld, Bielefeld, Germany; 3 Leibniz Research Centre for Working Environment and Human Factors, Dortmund, Germany

## Abstract

The brain needs to identify redundant sensory signals in order to integrate them optimally. The identification process, referred to as causal inference, depends on the spatial and temporal correspondence of the incoming sensory signals (‘online sensory causality evidence’) as well as on prior expectations regarding their causal relation. We here examine whether the same causal inference process underlies spatial integration of actions and their visual consequences. We used a basic cursor-control task for which online sensory causality evidence is provided by the correlated hand and cursor movements, and prior expectations are formed by everyday experience of such correlated movements. Participants made out-and-back movements and subsequently judged the hand or cursor movement endpoints. In one condition, we omitted the online sensory causality evidence by showing the cursor only at the movement endpoint. The integration strength was lower than in conditions where the cursor was visible during the outward movement, but a substantial level of integration persisted. These findings support the hypothesis that the binding of actions and their visual consequences is based on the general mechanism of optimal integration, and they specifically show that such binding can occur even if it is previous experience only that identifies the action consequence.

## Introduction

Part of the sensory input that the brain receives is redundant, meaning that the signals provide information on the same property of a physical object (e.g., the felt and seen size of a hand-held object) or event (e.g., the auditory and visually indicated location of a person speaking to you). Redundant sensory information can be integrated in an optimal manner, which entails a weighted average of the unisensory information with weights proportional to their relative reliability (i.e., inverse variability)^1–3^. The thus integrated perceptual estimate is referred to as ‘optimal’ because its reliability is higher than that of the unisensory estimates. Reliability can be further increased by integrating the sensory information with prior knowledge, often referred to as Bayesian inference in perception e.g.^4,5^. In case of discrepancies between the unisensory signals in the spatial or temporal domain, integration gives rise to mutual biases in the bimodal estimates (i.e., the estimate of ‘a’ in the presence of ‘b’, and vice versa) cf.^6,7^. The stronger the integration, the stronger these biases are and the harder it is to discriminate the spatial or temporal characteristics, in particular when the discrepancies are only small^8^. These ‘costs’, the biases in particular, are useful tools in studying integration.

**Figure 1:**
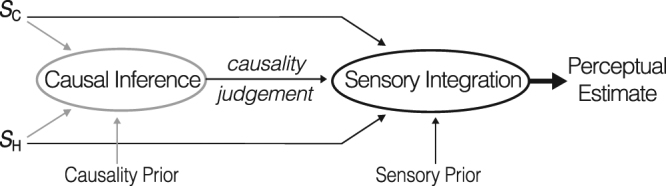
Causal inference and sensory integration. The schematic illustration shows that two redundant sensory signals (S_C_ and S_H_) can be combined into a single perceptual estimate through the process of optimal sensory integration. If prior sensory information is included as well, the integration process if referred to as Bayesian. Yet before integrating, the brain has to derive whether or not the two signals are redundant. The responsible causal inference processed is based on the content (in particular the spatial and temporal similarity) of the sensory signals (the *online* causality evidence). Causal inference can also be influence by prior causality expectations and knowledge, and it therefore a Bayesian process as well. The outcome of the causal inference process, the causality judgment, is an input to the integration strength where it influences the integration strength.

Integration of redundant sensory information presupposes identification of those sensory signals among the wealth of incoming sensory information. This process is referred to as causal inference because redundant signals usually have a common underlying cause (e.g., a physical object or event). Causal inference is believed to be a Bayesian process as well, meaning that it is based on causality information inherent to the sensory signals themselves (here referred to as *online sensory causality evidence*) as well as on causality information provided by prior experience and knowledge (here referred to as *prior causality expectation*)^9–11^. The outcome of causal inference in general is not an all-or-nothing estimate, but rather a continuous measure of the ‘belief’ that two – or more – sensory signals ‘belong together’. The outcome of causal inference, the causality judgment, can be conceived of as an input to the subsequent integration process, where it determines the integration strength^12–16^. Figure 1 schematically illustrates the dependency of causal inference on online sensory evidence and prior causality expectations and its influence on sensory integration. Also included is the influence of sensory priors on the perceptual estimates.

Online sensory causality evidence is provided by the correspondence of the sensory signals, in particular their spatial co-location, temporal co-occurrence, and structural similarity (e.g., cross-correlation)^17–20^. Prior causality expectations – or the strength of the ‘unity assumption’ that different sensory signals come from the same object or event - can be of various origins, for a review see^21^. A powerful factor is the relation between sensory signals as experienced over previous encounters. This is nicely illustrated by a study in which participants were trained with an artificial relation between an object’s luminance and its stiffness over a one-hour experimental session^22^. After this training, participants’ discrimination performance was reduced, indicating that participants started integrating these sources of information despite the absence of causality evidence in the incoming signals themselves. Thus, prior expectations can suffice to induce sensory integration.

Multisensory integration and causal inference have mainly been studied with purely perceptual tasks, whereby the sensory stimuli are related to an object or event in the external world. Popular examples are the ventriloquism effect and the McGurk effect^21^. However, the appraisal of sensory causal relations is essential not only for the identification of redundant information in purely perceptual tasks, but also in linking one’s own actions to their sensory consequences in sensorimotor tasks. In order to operate a mouse on a computer monitor, for example, the brain needs to estimate which of the incoming visual signals relates to the action performed by the hand. The brain is able to do this successfully, as evidenced by our ability to operate a computer via the cursor. A previous study found differences in the processing visual information regarding target or a cursor, and suggested that the brain establishes a special link between hand-position and cursor-position information based on the principles of optimal sensory integration^23^.

There is some initial evidence for this hypothesis from studies using basic cursor-control tasks in which spatial discrepancies were created between the hand and cursor movement directions and thus in their movement end positions. It was found that participants show strong mutual biases in their judgments of these hand and cursor end positions^24–29^. These spatial biases bear some similarity to the temporal biases known as ‘intentional binding’^30^. We recently demonstrated that the spatial biases in cursor-control scale with the reliability of the unisensory hand and cursor position estimates, consistent with optimal integration model predictions^24^. Moreover, we found that the integration strength declined when the spatiotemporal hand-cursor cross-correlation during each movement was reduced by non-linearly transforming the cursor trajectory^25^. Thus, these findings indicate that the brain integrates spatial information regarding an action (here of the hand) with spatial information regarding its visual consequence (here the motion of the cursor), and that spatiotemporal kinematic correlations provide online sensory causality evidence for this integration.

The present study is a further test of the hypothesis that binding of sensory information on actions and their visual effects is based on the general mechanisms of optimal integration. Specifically, we test whether the previously observed integration is based on both online sensory causality evidence and on prior causality expectations, thus following the fundamental Bayesian principle. In a familiar sensorimotor task, like cursor-control, such a prior basically comprises the expectation that the cursor does exactly what the hand does. In absence of online sensory evidence that causally relates the cursor motion to the hand movement (i.e., the hand-cursor correlated trajectories), the integration of hand and cursor position information is thus predicted to remain present – albeit to a reduced degree – based on previous experience of causally related hand and cursor movements. The current experiment was designed to test this prediction. We used a cursor-control task in which participants made out-and back hand movements after which they judged the most outward position of the hand or the cursor. In the critical condition, the cursor was only shown in this end position and not during the dynamic part of the outward movement, meaning that sensory evidence of hand-cursor kinematic cross-correlations during each movement was absent. We examined the strength of the integration and made comparisons between the observed characteristics of the position judgments and those predicted for optimal integration.

## Results

**Figure 2:**
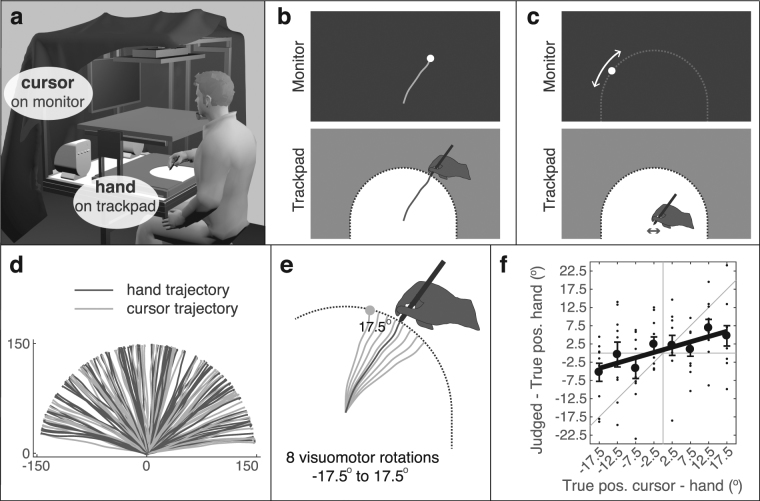
Experimental setup and rationale. (**a**) Participants moved their right hand on a trackpad in the horizontal plane. Hand and trackpad were occluded by a screen. The corresponding movement of a cursor (6-mm diameter light grey dot) could be shown on the monitor in the frontoparallel plane. (**b**) The hand movements were constrained to a half-circular workspace, the boundary of which is referred to as the stopper ring (dotted line). The outward hand movement (i.e., from the center to the stopper ring) could be accompanied by the corresponding cursor motion. For illustration purposes we illustrated the cursor trajectory here as the white line and we show the cursor a bit larger than the correct scale. (**c**) After returning to the remembered start position, participants judged the remembered final hand or cursor position. They did so by moving a dot, similar appearance as the cursor, over an invisible track that corresponded to all possible movement endpoints. (**d**) The movement endpoints were spread out over the full range of the stopper ring. The trajectories here are from a representative participant (*BiHand* trials in condition *DynEnd*). (**e**) The direction of the cursor movement was manipulated with 1 out of 8 possible visuomotor rotation magnitudes, thus causing a discrepancy between the hand and cursor movement endpoints. (**f**) The relative bias in the hand position judgments toward the cursor position were obtained by regressing the angular difference between the judged and true hand positions on the angular difference between the true cursor and hand positions. The slope of the regression line quantifies the relative bias; the variance over the residuals quantifies the variability of the position estimates. The bias in the cursor position judgment to the hand position was computed analogously. The data shown here correspond to the trajectories shown in panel d.

In the experiment, participants made out-and-back movements with a stylus in their unseen right hand on a trackpad in the horizontal plane. The corresponding motion of a cursor was displayed on a monitor in the frontoparallel plane (see Fig. 2a). Participants started their movement at the center of a half-circular workspace, moved fluently to its boundary, and upon hitting the boundary they immediately returned to the remembered start position. Figure 2b illustrates an outward trajectory. Directly after the backward movement they were asked to judge the movement endpoint (i.e., the position on the workspace boundary) of either their hand or the cursor (see *Methods* for details). The trials in which the hand endpoint was judged are referred to as *BiHand* trials; those in which the cursor endpoint was judged are referred to as *BiCursor* trials. Participants indicated their position judgments on the monitor, meaning that the hand position judgment required a coordinate transformation to the frontoparallel plane. Position judgment were provided by the participants making small left-right movements with the stylus on the tablet, which caused a small dot to move over the monitor along a circular path defined by all potential end points of the outward movements (see Fig. 2c).

To prevent stereotypical movements and position judgments, each trial started with a symbol on the monitor that instructed participants in which direction they should approximately move. Movement endpoints were thus scattered across the workspace (see Fig. 2d for an exemplary participant). Critically, the cursor movement was slightly rotated relative to the hand movement, with a rotation magnitude varying randomly between −17.5° and 17.5° in steps of 5°. This visuomotor rotation created a small – generally unnoticed – discrepancy between the hand and cursor endpoints (see Fig. 2e). We assessed the sensory integration for each participant and experimental condition by regressing the angular error of the position judgment in each trial (with 10 repetitions per visuomotor rotation) on the true hand-cursor angular discrepancy. The slope of the regression quantifies the perceptual bias as a proportion of the rotation (i.e., the relative error). The variance of the perceptual judgments is estimated by the variance of the residuals. Finally, integration strength is quantified by the sum of the biases for the hand position judgments (*BiHand* trials) and cursor position judgments (*BiCursor* trials). Figure 2f illustrates the regression of the hand position judgment errors for an exemplary participant.

### Effect of online causality evidence on the integration strength

**Figure 3:**
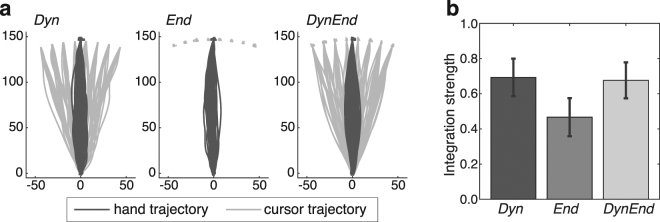
Visibility conditions and integration strength. (**a**) The visibility of the cursor was manipulated according to three visibility conditions. For illustration purposes we here rotated all trajectories to align the hand endpoints at 90 degrees. Condition *Dyn*: the cursor was visible during the dynamic phase of the outward hand movement. Condition *End*: the cursor was visible only when the hand was at the movement endpoint. Condition *DynEnd*: the cursor was visible both during the dynamic phase and at the endpoint. (**b**) The integration strength for the three visibility conditions. Any value between 1 (sensory fusion) and 0 (sensory independence) indicates partial integration.

We manipulated the availability of online sensory causality evidence by showing the cursor at selective parts of the hand movement trajectory. The cursor was shown either: (*i*) only during the dynamic part of the outward hand movement, that is, until the hand reached the endpoint (condition *Dyn*), (ii) only at the endpoint, meaning that there was no simultaneous hand and cursor movement and hence no online sensory causality evidence during each movement (condition *End*), or (iii) both dynamically and at the endpoint (condition *DynEnd*). Figure 3a illustrates these conditions using the movement trajectories of an exemplary participant.

We found a reduced integration strength in condition *End* as compared to the two conditions with dynamic cursor-position information, *Dyn* (*t*_(11)_ = 4.26, *p* = 0.001, *d* = 1.23) and *DynEnd* (*t*_(11)_ = 2.61, *p* = 0.024, *d* = 0.75) (see Fig. 3b). There was no difference in integration strength between the two conditions with dynamic cursor-position information (*t*_(11)_ = 0.27, *p* = 0.796, *d* = 0.08), suggesting that there was no additional causality information in the simultaneously present hand and cursor position information when they were static at the endpoint.

### Position judgments – unisensory variability

Our experiment was designed to examine the effect of omitting online sensory causality evidence on the strength of hand-cursor sensory integration. This experimental manipulation, however, could in principle have additional influences on the process of sensory integration, such as introducing biases in addition to those expected for optimal integration. To disentangle these effects, we analyzed the characteristics of the bimodal position judgments against optimal integration model predictions as a benchmark. These model predictions required the integration strength and the variances of the unisensory position estimates as input (see next paragraph). We therefore included two unimodal trial types: *UniHand* trials (participants made the out-and-back hand movement without seeing the cursor) and *UniCursor* trials (participants did not make a hand movement but saw the cursor moving).

**Figure 4:**
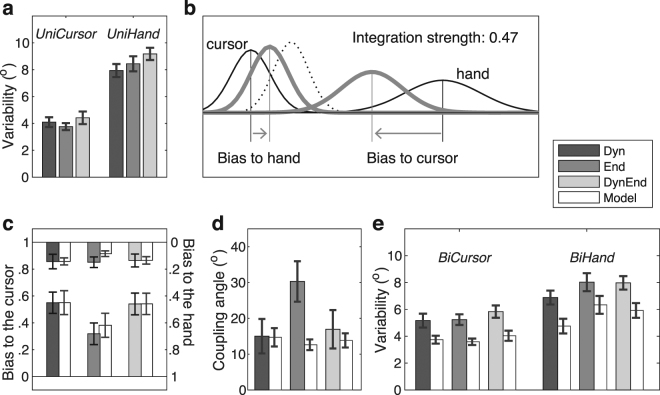
Observed and predicted characteristics of the position judgments. (**a**) The average variability (i.e., standard deviation) of the unisensory position judgments for the three visibility conditions. (**b**) Illustration of partial integration. The unisensory signals (thin black lines) are weighted according to their reliability (i.e., inverse variability), whereby for fusion (black dotted line) the weights add up to one and for partial integration they sum up to a value between 0 and 1, which quantifies the integration strength. (**c**) The average biases in the bimodal position judgments as observed (grey bars) and predicted (accompanying open bars). The biases in the hand position judgments toward the cursor position (*BiHand* trials) are indicated by the standing bars; the biases in cursor position judgment toward the hand position (*BiCursor* trials) are indicated by the hanging bars. (**d**) The coupling angle indicates the asymmetry of the position biases, with higher values indicating a stronger bias to the hand position. The observed coupling angles (grey bars) are accompanied by the predicted angles (open bars). (**e**) The observed (grey bars) and predicted (open bars) variability of the bimodal position judgments for the three visibility conditions as quantified by the standard deviations.

The standard deviations of the unisensory position judgments are shown in Fig. 4a. We found that judgments of hand endpoints were more variable than judgments of cursor endpoints. The visibility conditions did not significantly affect the variability in *UniCursor* trials as indicated by pairwise paired-samples *t*-tests (all *p*-values > 0.091), suggesting that seeing the outward cursor movement did not contribute to judging its static endpoint. In the *UniHand* trials the variability was slightly modulated across conditions. The difference between conditions *Dyn* and *DynEnd* was significant (*t*_(11)_ = 2.86, *p* = 0.016, *d* = 0.82), the others were not (*p*-values > 0.121). This effect was unexpected given that these trials were identical in the three visibility conditions in that no cursor was shown. It thus most likely reflects a context effect of neighboring trials or it is a chance result. A context effect is possible because in condition *DynEnd* visual feedback was available for the longest time, as compared with the other conditions, which could have been associated with a generally reduced attention to hand-position information.

### Partial integration model predictions

We derived model predictions for optimal integration using a model, referred to as the Coupling Prior model, that allows for partial integration at any strength e.g.^2,12,24,31^. The model is an extension of the classic optimal integration model that predicts fusion of two sensory estimates (see dotted line in Fig. 4b). Sensory fusion is a weighted average of the two unisensory estimates, whereby the weights reflect the unisensory reliabilities and their sum equals 1. Partial integration as described by the coupling model is a similar weighted average of the two unisensory estimates with weights that sum up to less than 1; the sum of the weights indicates the strength of the integration. Based on two unisensory variances and the observed integration strength, the model predicts the biases (i.e., the weights) and the variances that correspond to optimal partial integration. This is illustrated in Fig. 4b by two unisensory estimates (black solid lines) with variabilities that correspond to the average unisensory hand and cursor variabilities of Fig. 4a, and the bimodal estimates (grey thick lines) that correspond to the average integration strength observed in condition *End*. The black dotted line indicates the bimodal prediction for an integration strength of 1 (i.e., full fusion).

### Position judgments – bimodal biases

The observed and predicted bimodal biases of hand-position and cursor-position judgments are illustrated in Fig. 4c by the filled and open bars, respectively. The standing bars indicate the bias in the hand position judgments toward the cursor (*BiHand* trials); the hanging bars indicate the bias in the cursor position judgments toward the hand (*BiCursor* trials). For the *BiCursor* trials, the bias was similar for the three visibility conditions. For the *BiHand* trials, the bias was substantially smaller in condition *End* as compared to the two conditions with dynamic cursor-position information. The observed biases closely matched the partial integration model predictions. A 2 (data vs. model) × 3 (conditions) ANOVA for the *BiCursor* trials revealed no significant main or interaction effects (all *p*-values > 0.170). For the *BiHand* trials a corresponding ANOVA revealed only a main effect of condition (F_2,22_ = 7.13, *p* = 0.004), reflecting the lower cursor weight in condition *End*. Pairwise comparisons revealed that condition *End* differed from condition *Dyn* (*t*_(11)_ = 3.72, *p* = 0.003, *d* = 1.07) and condition *DynEnd* (*t*_(11)_ = 2.56, *p* = 0.027, *d* = 0.74), but the difference between the latter two conditions was not significant (*t*_(11)_ = 0.19, *p* = 0.853, *d* = 0.05). The six correlations between the predicted and observed biases for *BiHand* and *BiCursor* trials were significant in all three visibility conditions (all *p*-values < 0.006), with correlations ranging between 0.74 and 0.96. This indicates that participants’ behavior was consistent with model predictions not only with respect to means, but also with respect to inter-individual variability.

Although not statistically significant in the ANOVAs, visual inspection of the observed and predicted biases suggests that the biases in condition *End* might be drawn toward the hand position slightly more than predicted. For a more focused examination we analyzed the observed and predicted integration asymmetry (see Fig. 4d). This measure expresses the ratio of the biases in the bimodal hand and cursor position judgments on an angular scale (i.e., the coupling angle) with 0° indicating a pure bias toward the cursor position and 90° a pure bias toward the hand position. The coupling angle has the benefit of being independent of individual differences in coupling strength, yet the disadvantage of being susceptible to noise in the measures especially for low integration strengths. For this latter reason we excluded the data of three participants with integration strengths less than 0.2 in at least one of the three experimental conditions. We found no significant difference between the observed and predicted coupling angles in conditions *Dyn* and *DynEnd* (*p* > 0.520), and a higher-than-predicted coupling angle for condition *End* (*t*_(11)_ = 3.13, *p* = 0.014, *d* = 1.04). Thus, the limited cursor visibility in condition *End* went along with an additional judgment bias towards the hand position that could not be attributed to the purely reliability-based weighting mechanism of optimal integration.

### Position judgments – bimodal variability

The observed and predicted bimodal standard deviations are shown in Fig. 4e by the filled and open bars, respectively. The variabilities of the judgments in *BiCursor* and *BiHand* trials were in-between those in the *UniCursor* and *UniHand* trials. Although this finding is consistent with our previous findings^24,25^, it is clearly at odds with model predictions for (partial) integration because integration always leads to a reduction of the bimodal judgment variability as compared to the variability of the corresponding unisensory judgments. Figure 4e also shows a modulation of the variability across the three conditions that was quite consistent across the observed and predicted variabilities. An analysis of the differences between observed and predicted standard deviations by means of a 3 (conditions) × 2 (*BiCursor* vs *BiHand*) ANOVA revealed no significant effects (all *p*-values > 0.312). The similarity between predicted and observed standard deviations was furthermore indicated by significant correlations (*p*-values < 0.035) ranging between 0.60 and 0.92, and one marginally significant correlation (*r* = 0.56; *p* = 0.056) for *BiHand* trials in condition *Dyn*.

### Movement durations

The three visibility conditions differed with respect to the movement phase where the cursor was visible. To be able to assess possible effects of the concomitant differences in the duration of cursor visibility, we analyzed the duration of the outward movement and the amount of time that participants kept their hand at the endpoint. In this analysis we included only the bimodal trials. The mean durations of the outward movement were 1.26 ± 0.10 s (*Dyn*), 1.20 ± 0.12 s (*End*), 1.29 ± 0.15 s (*DynEnd*). Pairwise paired-samples *t-*tests indicated no significant differences (all p-values > 0.632). The average time at the endpoint was 0.45 ± 0.05 s (*Dyn*), 0.63 ± 0.06 s (*End*), 0.53 ± 0.06 s (*DynEnd*). The difference between condition *End* and condition *Dyn* was marginally significant (*t*_(11)_ = 2.05, *p* = 0.065, *d* = 0.59); the other comparisons were not (*p*-values > 0.280). Overall, there were clear differences with respect to the total time during which cursor position information was available between conditions *Dyn* (1.26 s), *End* (0.63 s), and *DynEnd* (1.82 s). We performed regression analyses for each visibility condition in order to assess the relation between the duration of the cursor visibility and the coupling strength. None of the three correlations was significant (all *p*-values > 0.243), suggesting that, at least within each visibility condition, the duration of cursor visibility had no influence on the strength of the sensory integration.

## Discussion

Optimal sensory integration presupposes the preceding identification of whether certain signals are redundant or not. Clearly, integration is beneficial for redundant signals related to the same attribute of a single object or event. However, sensory integration has also been observed for sensory signals related to one’s own actions and their sensory consequences, in particular for spatial estimates regarding the hand and the cursor in a cursor-control task e.g.^26–29^. In this task the integrated sensory signals relate to different objects, but to the same attribute, namely their position, for which there is a systematic relation as indicated by correlated movement trajectories. This is in contrast to the unrelated positions and trajectories of arbitrarily chosen different objects. We here addressed the general hypothesis that the binding of hand-position and cursor-position information obeys the general principles of optimal sensory integration^23^. More specifically, we hypothesized that the integration should be based on the same causal inference process for estimating sensory redundancies as observed for the integration of attributes of singles object or events. That is, the integration should be based on both online sensory causality evidence and on prior causality expectations.

We used a cursor-control task in which participants judged the hand or cursor endpoint of an outward movement. We manipulated the availability of online sensory causality evidence – the hand-cursor kinematic cross-correlations^25^ – by providing or omitting visual information of the cursor during the dynamic part of the hand movement. Consistent with previous studies, we observed partial integration as indicated by the typical biases in the hand and cursor position judgments^24–29^. With only endpoint visual feedback, the strength of sensory integration was less than in the two conditions with dynamic visual feedback, yet clearly above zero. This finding indicates that causal inference is not exclusively based on online sensory causality evidence, that is, on the hand-cursor cross-correlations as experienced in each individual trial immediately preceding the judgments, but also on prior causality expectations that are likely based on preceding experience of sensory cross-correlations during everyday computer use. We thus conclude that integration of hand position and cursor position in a cursor-control task is based both on online sensory causality evidence and prior causality expectations.

Our conclusion is based on the supposition that there is no online sensory causality evidence in the absence of visual feedback during the dynamic phase of the movement. This assumption was motivated by the finding that kinematic cross-correlations affect the strength of integration in the cursor-control task^25^ and that other known sources of sensory causality evidence (e.g., spatial co-location) are absent e.g.^10,11^. However, the assumption can be challenged because of the temporal coincidence of the visual presentation of the endpoint with the end of the outward hand movement which in principle could provide online sensory causality evidence.

A comparison of the conditions with dynamic-plus-endpoint feedback (*DynEnd*) and with only dynamic feedback (*Dyn*) allows an assessment of the causality evidence provided by the simultaneous presentation of cursor and hand position at the endpoint: there was no difference in integration strength between these two conditions. This finding strongly suggests that no additional online sensory causality evidence was provided by showing the cursor at the endpoint of the movement. This was the case even though overall the duration of cursor visibility was longer in condition *DynEnd* than in condition *Dyn*, but for the additional duration there was essentially no (joint) variation of hand and cursor positions. (The absent effect of the duration of cursor visibility was confirmed by the lack of correlations between individual integration strengths and individual durations of cursor visibility.) We thus attribute the reduction in integration strength in the condition with only endpoint visual feedback to the absence of the sensory cross-correlations as the source of online sensory causality evidence, and the remaining integration strength to the prior causality expectations.

Our data suggest that participants slightly adjusted their behavior to the limited availability of visual cursor position information in condition *End* (endpoint visual feedback only). First, we found that participants rested their hand at the end position slightly longer in this condition than in the dynamic feedback condition. Possibly, participants made these movement adjustments aiming to optimize the reliability of their sensory estimates^32^. In this case one could expect reduced variability of the unimodal judgments of cursor position in condition *End*, which was not observed in our data (at least not significantly). However, the potential positive effect of prolonged time at the endpoint might have been just sufficient to compensate a potential negative effect of the reduced cursor visibility just before the endpoint was reached. This cannot be assessed from our current data, as it would require experimenter-controlled durations of cursor visibility at the endpoint. Second, we found that the reduced integration strength in condition *End* went along with a change increased asymmetry of the biases toward the hand position. This change in asymmetry was not predicted by the optimal integration model, which predicts the asymmetry purely from the reliabilities of the individual estimates of hand and cursor positions. It thus indicates that, in addition to reliability-based weighting, the brain seems to adjust the sensory weights depending on context^24,33–35^ – in this case an increased emphasis on the hand position information due to the limited availability of visual information.

The observed unimodal variabilities and the observed integration strengths formed the basis for the optimal integration model predictions of the bimodal variabilities. The predicted modulation of the variability across the visibility conditions was similar to the observed modulation. In particular, the reduced coupling strength in condition *End* resulted in the predicted increased variability in the bimodal hand position judgments, and the increased unimodal hand position variability in condition *DynEnd* resulted in the predicted increased bimodal variability in both hand and cursor position judgments. At least as striking as the similarity in the modulation across visibility conditions was the clear offset between the observed and predicted variability. Most notably, the variability of cursor-position judgments was larger in bimodal trials than in unimodal trials. This violates a basic prediction of optimal multisensory integration, namely that integration serves to reduce the variability of both the more and the less reliable estimate and not only the reliability of the less reliable one. For the hand-position judgment, we did see a reduction of the bimodal variability compared to the unisensory variability. This excludes that the data would be better explained by a strategy that doesn’t involve any integration, such as a switching strategy. We have seen the overall discrepancy between observed and predicted standard deviations before and have discussed some possible origins in more detail elsewhere^24,25^. A plausible explanation is that an additional component of variability is currently not adequately captured by the model, such as perhaps the transformation noise arising from to the change in reference frame (horizontal to vertical) or modality (hand to cursor position) in this task.

A difference between integration in purely perceptual tasks and in a sensorimotor task, like cursor-control, is the potential involvement of predicted sensory consequences obtained from forward models e.g.^36,37^. These predicted sensory consequences can substitute missing sensory information in the integration process, as was shown for example by expert drummers being able to detect audiovisual asynchrony of another person drumming when visual information was omitted well before drumstick-drumhead impact^38^. Can predicted sensory consequences also enter the process of causal inference? In the case of the current cursor-control task, this would imply that observers integrate information on hand position with predicted information on cursor position, even if the cursor is invisible during the outward trajectory (thereby omitting the causality information) as well as at the endpoint (the to-be-judged visual position). This does not seem very plausible.

For the sensory integration of hand positions and their visual effects, cursor positions in particular, the current study reveals that partial integration can persist in the absence of online sensory causality evidence, based on prior causality expectations. The notion of a *causality prior* complements a large body of literature showing that the brain forms *sensory priors* from previous sensory experience, both in purely perceptual tasks and in sensorimotor control^39–43^ (see Fig. 1). Such sensory priors capture the *a priori* belief regarding the magnitude of a certain to-be-estimated parameter, such as a prior for object constancy or color constancy cf.^4^, and act as additional sensory input in the integration process. Causality priors, in contrast, are concerned with the *a priori* belief regarding common or different causes of sensory signals and they influence the strength of the integration. For sensory priors it has been shown that they can be flexibly adjusted to altered sensory regularities^42–44^ (e.g., a systematic change in an object’s average movement speed). With respect to the prior causality expectations, it remains to be determined how flexibly they can be adjusted.

## Methods

### Participants

The experiment was conducted in accordance with the declaration of Helsinki and approved by the Bielefeld University Ethics Committee. Twelve right-handed participants (aged 20 to 26 years; 9 female) volunteered to take part in the experiment. We ensured that the participants were experienced computer users by recruiting them from the Bielefeld University student pool. All participants gave written informed consent and were compensated with a payment of €6 per hour.

### Apparatus

The experimental setup is illustrated in Fig. 2a. Participants sat at a table with a digitizer tablet on top of it (Wacom Intuos4 XL; 48.8 by 30.5 cm). A chinrest supported them in keeping a 60 cm viewing distance from a computer monitor (Samsung MD230; 23 inch; 50.9 by 28.6 cm). They held a digitizer stylus in their right hand and pressed a button on the stylus with their thumb or index finger when required. A semi-circular workspace of 15 cm radius was created on the tablet by means of a 5 mm thick PVC template. This template provided a mechanical stop for the outward movements and is referred to as the “stopper ring”. Direct vision of hand and stylus was prevented by a horizontal opaque board. The position of the stylus was recorded (at 60 Hz) and mapped online to the position of a (visible or invisible) cursor on the monitor, using MATLAB with the Psychophysics Toolbox extension^45^. During the experiment, the room was dark such that only the images on the monitor were visible. All images were presented in light grey on a black background.

### Task

Participants performed out-and-back movements on the digitizer tablet, from the center of the semicircular workspace until hitting the stopper ring and then immediately back to the remembered start position. Visual feedback was provided by a cursor (a 6-mm light grey dot) on the computer monitor. Depending on the experimental condition (see *Visibility conditions*), the cursor was visible during different parts of the outward hand movements (see Fig. 3a). After each out-and-back movement, the word ‘HAND’ or ‘CURSOR’ appeared on the monitor to instruct participants to report the memorized final position of, respectively, the hand or the cursor (i.e., where they had hit the stopper ring). 500 ms later, a visual marker (6-mm diameter white dot) appeared at the far left or far right side of an invisible semi-circular track covering all possible endpoints of the outward movements (i.e., stopper ring in monitor space). Participants moved the marker along the track by making small movements to the left or right (<1 cm amplitude) with the stylus on the tablet (see Fig. 2c). Once satisfied with the marker’s position, they confirmed their judgment by pressing the stylus’ button. There were no time constraints in making these position judgments.

The direction of cursor motion was always rotated relative to the direction of hand movement, as is illustrated in Fig. 2e. The thus created discrepancy between the physical hand and cursor endpoints was needed to quantify the biases in the position judgments. The visuomotor rotation varied randomly across trials between −17.5° and +17.5° in steps of 5°, with a mean of 0°. Participants were not informed about the visuomotor rotation (they were told that the experiment was about the effect of attention on remembered sensations) and none of the participants reported noticing it in a structured post-experimental interview.

**Figure 5:**
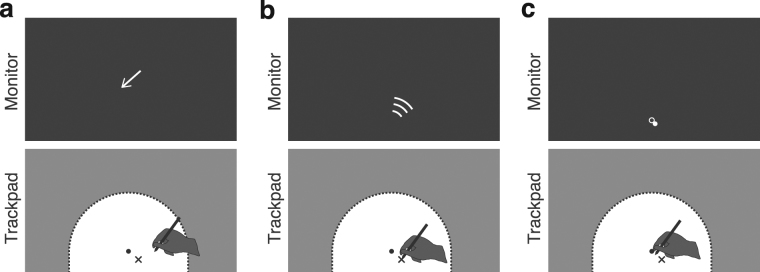
Procedure before movement onset. (**a**) An arrow operated as a compass to guide the participant’s hand to an initial position (indicated here by the back cross) that was just below the start position (the black dot). (**b**) A WiFi-like symbol was shown that indicated participants in which direction they should approximately make their outward hand movement. The symbol’s color indicated the subsequent trials type: *UniCursor* (red – indicating that they shouldn’t move their hand), *UniHand* (green), or one of the two bimodal ones (light grey). (**c**) The cursor (light grey dot) and start position (open circle) where shown to guide participants to the start position. Once they reached the start position, it disappeared. Depending on the condition, the cursor either remained visible or it disappeared as well.

Each individual trial started with a short procedure in which the hand was guided to the start position (see Fig. 5). First, a compass was shown that guided participants to an initial position (6 mm diameter light grey dot) that was defined as a point 1 to 2 cm below, and −2 to 2 cm beside the center position. Second, a light grey WiFi-like symbol was presented on the monitor. This symbol instructed participants to move in the indicated approximate direction, and its color instructed participants on the type or trial: bimodal (light grey), green (*BiHand*), or red (*BiCursor* – meaning don’t move the hand). The direction instruction was included to prevent stereotyped outward movements and hence stereotyped position judgments. There were eight instructed approximate directions, centered at −56° to 56° relative to straight-ahead, in steps of 16°. The order of their presentation was semi-randomized (see *Design*). The resulting movement directions were scattered over the entire 180° workspace (see Fig. 2d). Third, both the cursor (6-mm diameter filled dot) and the start position (7-mm diameter open dot) were shown. The start position disappeared once participants reached it. The cursor either remained visible (condition *Dyn* and *DynEnd*) or disappeared (condition *End*) for the subsequent outward movement.

### Visibility Conditions

We tested three conditions that differed with respect to when the cursor was visible (see Fig. 3a). In the dynamic condition *(Dyn)*, the visual feedback was presented during the outward movement, beginning at the start position and ending at a threshold of 97% of the 15 cm distance to the stopper ring. In the endpoint condition *(End)*, the cursor was presented only at the very end of the hand movement, becoming visible at the 97% threshold and disappearing when the hand crossed this threshold again during the backward movement. Due to the thickness of the stylus, and depending on the exact posture of the stylus in the hand, participants moved their hand between 1.1 mm and 4.8 mm (average 3.1 mm) while endpoint feedback was presented. They thus had minimal exposure to correlated hand and cursor movements in this condition. Finally, in the third experimental condition both dynamic and endpoint visual feedback were provided (*DynEnd*).

### Trial types

In the bimodal trials, the outward hand movement was accompanied by visual feedback of the movement and/or its endpoint, depending on visibility condition. After participants returned their hand to the center position they judged either the final hand position (*BiHand* trials) or the final cursor position (*BiCursor* trials), according to the instruction on the monitor (see *Task*). In one unimodal trial type, participants made the outward hand movement without any visual feedback, after which they judged the final hand position (*UniHand* trials). In the other unimodal trial type (*UniCursor* trials), participants kept their hand static at the center position while the cursor was visible during an outward movement and/or at the endpoint (depending on the visibility condition). In these trials the cursor motion and/or endpoint was a replay of a cursor trajectory and/or endpoint recorded in a preceding *BiCursor* trial. Participants were instructed to judge the final cursor position after a delay that was equal to the average duration of the return movements in the preceding trials.

### Design

The experimental trials differed with respect to 3 visibility conditions, 4 trial types, and 8 visuomotor rotations (the visuomotor rotation was a dummy variable in the unimodal trials). The three visibility conditions were assigned to separate experimental sessions, and the order of these conditions across sessions was counterbalanced across participants. For each visibility condition, the set of 32 trials (4 trial types × 8 visuomotor rotations) was repeated 10 times, resulting in a total of 320 trials per session. Per repetition set, the order of the 32 trials was semi-randomized with the constraint that each *UniCursor* trial occurred later in the sequence than the corresponding *BiCursor* trial (because the cursor trajectory presented in the *UniCursor* trials was recorded in the corresponding *BiCursor* trial). For the trials of each repetition set we randomly allocated one of the eight visuomotor rotations to one of the eight approximate instructed movement directions, thus preventing a systematic relation between movement direction and rotation. Each experimental session was preceded by up to 28 familiarization trials, during which the experimenter gave verbal instructions on the required task execution. The familiarization ended when participants were able to correctly perform the task. We organized the total number of 320 trials per session (without familiarization) into 6 blocks with short breaks in-between. Each session was performed on a separate day and took about 2.5 hours to complete.

### Data collection and outlier analysis

In each trial we recorded the trajectories of hand and cursor movements as well as the judged position of cursor or hand. Our main interest was in the physical positions of cursor and hand at the end of the outward movement (defined as positions when the outward movement had reached the threshold of 97% of the distance to the stopper ring) and the judged position of cursor or hand (defined as positions of the visual marker when the participants pressed the stylus’ button). These positions were converted into angles of a polar coordinate system with the origin in the movements’ start position (i.e., the center position of the workspace); the distance of these positions from the origin of the polar coordinate system was constant (i.e., the stopper ring radius) and therefore not analyzed.

Before computing the dependent variables, we screened the data for outliers and we corrected the judged positions for hysteresis effects resulting from the clockwise or anti-clockwise motions of the visual marker. Outliers were defined as: (1) trials in which participants moved their hand more than 2.5° along the stopper ring before returning to the center position, (2) trials in which the outward movement direction deviated more than 35° from the instructed direction, and (3) trials in which the angular deviation between the physical and judged end positions was larger than 35° (i.e., twice the maximal visuomotor rotation). For each participant, out of the 80 trials (8 rotations x 10 repetitions) per trial type and visibility condition, between 0 and 14 trials were excluded (mean criterion 1: 0.61 trials or 0.8%; mean criterion 2: 0.80 trials or 1.0%; mean criterion 3: 0.24 trials or 0.3%). Position judgments were potentially affected by hysteresis because the response task started with the visual marker at either the far left or far right of the semi-circular track. We computed for each visibility condition (i.e., 320 trials) the regression of the judged positions on the physical positions with the constraint of a single slope, but two different intercepts for responses starting left or right. We corrected the judged positions for the difference between the intercepts for the left and right start positions (which ranged from −5.0° to 1.5°) by adding or subtracting half the difference.

### Dependent variables

We compared the three visibility conditions in terms of the overall integration strength, the biases of judged cursor and hand positions, the standard deviations of the position judgments, and the asymmetry of the biases. The observed biases, standard deviations and asymmetry were compared with model prediction for partial optimal integration. Additionally, we determined the duration of the hand movements and thus the duration of the cursor visibility in the different conditions.

To derive the magnitude of the biases in the position judgments, we computed for each of the bimodal trial types the angular deviation between the judged and the physical hand or cursor positions (for *BiHand* and *BiCursor* trials, respectively). We then regressed, for each trial type and visibility condition separately, the angular deviations on the visuomotor rotations. This is illustrated in Fig. 2f. The slopes of these regressions indicate the proportional biases (i.e., the attraction of the judgments towards the other modality as proportions of the visuomotor rotation). The proportional biases or slopes correspond to weights assigned to hand-position and cursor-position estimates in sensory integration^24^ (see Fig. 4b). The slope in *BiHand* trials quantifies the weight *w*_C_obs_ given to the cursor’s end position, with *w*_C_obs_ = 0 indicating that the judged hand positions match the physical hand positions and *w*_C_obs_ = 1 indicating that they match the physical cursor positions. The slope in *BiCursor* trials quantifies the weight *w*_H_obs_ given to the hand’s end position, with *w*_H_obs_ = 0 indicating that the judged cursor positions match the physical cursor positions and *w*_H_obs_ = 1 indicating that they match the physical hand positions.

The variability of the position judgments was computed from the same regression analysis, more specifically, as the variance of the residuals. For this reason, the regressions were also computed for the unimodal trial types whereby the visuomotor rotation was a dummy variable. We thus computed the variances for each trial type: *UniHand* (σ^2^_H_obs_), *UniCursor* (σ^2^_C_obs_), *BiHand* (σ^2^_H.C_obs_), and *BiCursor* (σ^2^_C.H_obs_).

The integration strength λ_obs_ was computed as the sum of the observed weights in the bimodal position judgments as:

1 $${\lambda }_{{\rm{obs}}}={w}_{{\rm{H}}\_{\rm{obs}}}+{w}_{{\rm{C}}\_{\rm{obs}}}$$

An integration strength of 1 indicates full sensory integration (i.e., hand and cursor are judged to be in the same position). An integration strength of 0 indicates independence (i.e., hand and cursor positions are not biased toward each other). Any integration strength between 0 and 1 indicates partial sensory integration (i.e., a certain level of mutual biases in the hand and cursor position judgments). It should be noted that numerically the integration strength was not constrained to range between 0 and 1 because we determined *w*_H_obs_ and *w*_C_obs_ independently.

The asymmetry of the biases was defined from the observed weights in the bimodal position judgments as the *coupling angle* α_obs_:

2 $${{\rm{\alpha }}}_{{\rm{obs}}}={\rm{atan}}({w}_{{\rm{H}}\_{\rm{obs}}}/{w}_{{\rm{C}}\_{\rm{obs}}})$$

This coupling angle increases with the bias toward the hand position, with an angle of 45° indicating symmetric biases (i.e., *w*_H_obs_ = *w*_C_obs_). The coupling angle is independent of the integration strength and thus enables an assessment of the relative magnitude of the attraction biases across visibility conditions with differing integration strengths.

Last, we derived predictions for the biases, variability and asymmetry according to the Coupling Prior model proposed by Ernst e.g.^2,22,31^, for which the equation as they are provided here were derived by Debats and colleagues^24^. Note that the formal notion of the coupling prior here captures both the influences of online sensory causality evidence and prior causality expectations. The input to the model are the observed unisensory variances and the observed integration strength. In the model equations, the integration strength is converted into a parameter that is called coupling prior variance:

3 $${{\rm{\sigma }}}_{{\rm{prior}}}^{2}=\frac{1-{{\rm{\lambda }}}_{{\rm{obs}}}}{{\lambda }_{{\rm{obs}}}}({{\rm{\sigma }}}_{{\rm{C}}\_{\rm{obs}}}^{2}+{{\rm{\sigma }}}_{{\rm{H}}\_{\rm{obs}}}^{2})$$

The predicted optimal bimodal weights are then computed as:

4 $$\begin{array}{c}{w}_{H\_\mathrm{pred}}=\frac{{{\rm{\sigma }}}_{{\rm{C}}\_{\rm{obs}}}^{2}}{{{\rm{\sigma }}}_{{\rm{C}}\_{\rm{obs}}}^{2}+{{\rm{\sigma }}}_{{\rm{H}}\_{\rm{obs}}}^{2}+{{\rm{\sigma }}}_{{\rm{prior}}}^{2}},\,{\rm{and}}\\ {w}_{C\_\mathrm{pred}}=\frac{{{\rm{\sigma }}}_{{\rm{H}}\_{\rm{obs}}}^{2}}{{{\rm{\sigma }}}_{{\rm{C}}\_{\rm{obs}}}^{2}+{{\rm{\sigma }}}_{{\rm{H}}\_{\rm{obs}}}^{2}+{{\rm{\sigma }}}_{{\rm{prior}}}^{2}}\end{array}$$

The predicted optimal variances for the bimodal position judgments are computed as:

5 $$\begin{array}{c}{{\rm{\sigma }}}_{{\rm{C}}.{\rm{H}}\_{\rm{pred}}}^{2}=\frac{{{\rm{\sigma }}}_{{\rm{C}}\_{\rm{obs}}}^{2}({{\rm{\sigma }}}_{{\rm{H}}\_{\rm{obs}}}^{2}+{{\rm{\sigma }}}_{{\rm{prior}}}^{2})}{{{\rm{\sigma }}}_{{\rm{C}}\_{\rm{obs}}}^{2}+{{\rm{\sigma }}}_{{\rm{H}}\_{\rm{obs}}}^{2}+{{\rm{\sigma }}}_{{\rm{prior}}}^{2}},\,{\rm{and}}\\ {{\rm{\sigma }}}_{{\rm{H}}.{\rm{C}}\_{\rm{pred}}}^{2}=\frac{{{\rm{\sigma }}}_{{\rm{H}}\_{\rm{obs}}}^{2}({{\rm{\sigma }}}_{{\rm{C}}\_{\rm{obs}}}^{2}+{{\rm{\sigma }}}_{{\rm{prior}}}^{2})}{{{\rm{\sigma }}}_{{\rm{C}}\_{\rm{obs}}}^{2}+{{\rm{\sigma }}}_{{\rm{H}}\_\mathrm{obs}\,}^{2}+{{\rm{\sigma }}}_{{\rm{prior}}}^{2}}\end{array}$$

The predicted coupling angle was computed from the observed unimodal variances as:

6 $${\alpha }_{{\rm{pred}}}=\arctan ({{{\rm{\sigma }}}^{2}}_{{\rm{C}}\_{\rm{obs}}}/{{{\rm{\sigma }}}^{2}}_{{\rm{H}}\_{\rm{obs}}})$$

### Statistical Analysis

Statistical analyses were performed using ANOVA for repeated measures, paired-samples *t*-tests, and Pearson product-moment correlations. As dependent measure for the variability of the position judgments we used the *standard deviations* (σ) rather than the variances because their units (degrees of arc) can be more easily interpreted than the units of variances (squared degrees of arc). Numbers indicated in the text and figures refer to the mean ± standard error of the mean.

### Data availability

All data generated and analyzed in this study are available at pub.uni-bielefeld.de/data/2916617, with doi:10.4119/unibi/2916617.
